# 

**Published:** 1898-08

**Authors:** 


### CONTENTS:

### SELECTIONS.

Article I.—Practical Points on Porcelain Fillings. W. A. Capon, D. D. S ........ 145

" II.—Extraction While Swelling of Gums and Toothache... 150

" III.—How Shall we best insert a Gold Filling? Dr. Arthur Galusha Smith ........ 153

" IV.—Dental Ethics. Dr. F. J. Capon ........ 156

" V.—Adaptation and Retention of Dentures. A. O. Hunt, D. D. S ........ 163

" VI.—The Care of Children's Teeth. Dr. J. R. Lowe ........ 165

" VII.—The Extraction of Teeth. Dr. George B. Clements.... 169

" VIII.—Silver Nitrate in the Treatment of Pulpless Teeth. Dr. L. G. Noel ........ 172

" IX.—Germicides and Antiseptics in Dentistry. C. Bunting Colson, M. D., D. D. S ........ 175

### COMMUNICATED.

List of Committee's for the Joint Meeting of the Southern Branch of the National Dental Association and the Louisiana State Dental Society ........ 185

### MONTHLY SUMMARY.

The Carborundum Industry ........ 188

Alcohol as a Disinfectant ........ 190

Education and Starvation ........ 190

Hydronaphthol in the Treatment of Dental Pulps ........ 191

Prescription Writing ........ 191

A Dentsit ........ 192

